# Income loss after a cancer diagnosis in Germany: An analysis based on the socio‐economic panel survey

**DOI:** 10.1002/cam4.3913

**Published:** 2021-05-10

**Authors:** Diego Hernandez, Michael Schlander

**Affiliations:** ^1^ Division of Health Economics German Cancer Research Center (DKFZ) Heidelberg Germany; ^2^ Medical Faculty Mannheim Heidelberg University Mannheim Germany

**Keywords:** cancer diagnosis, financial burden, income loss, socio‐economic panel

## Abstract

**Background and Aims:**

Cancer treatments often require intensive use of healthcare services and limit patients’ ability to work, potentially causing them to become financially vulnerable. The present study is the first attempt to measure, on the German national level, the magnitude of absolute income loss after a cancer diagnosis.

**Methods:**

This study analyzes data from the Socio‐Economic Panel (SOEP) survey, one of the largest and most comprehensive household surveys in Germany, consisting of approximately 20,000 individuals, who are traced annually. The empirical strategy consists of ordinary least squares (OLS) and multinomial logistic estimators to measure changes in job income, work status, working hours, and pension as a result of reporting a cancer diagnosis for the period between 2009 and 2015. Sample consistency checks were conducted to limit measurement error biases.

**Results:**

Our results show that job incomes dropped between 26% and 28% within the year a cancer diagnosis was reported. The effect persisted for two years after the diagnosis and was no longer observable after four years. The finding was linked to an increased likelihood of unemployment and a reduction of working hours by 24%. Pension levels, on the other hand, were not affected by a cancer diagnosis.

**Conclusions:**

These findings suggest that many cancer patients are exposed to financial hardship in Germany, particularly when the cancer diagnosis occurs during their working age and before requirements to obtain a pension are met. Further research seems warranted to identify particularly vulnerable patient groups.

## Graphical abstract

The present study is the first attempt to measure, on the German national level, the magnitude of income loss after a cancer diagnosis. This study analyzes data from the Socio‐Economic Panel (SOEP) survey, one of the largest and most comprehensive household surveys in Germany, consisting of approximately 20,000 individuals, who are traced annually. Our empirical results suggests that many cancer patients are exposed to financial hardship in Germany, particularly when the cancer diagnosis occurs during their active life and before requirements to obtain a pension are met.

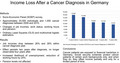

## 1 INTRODUCTION

Cancer has emerged as a leading cause of mortality worldwide. The World Health Organization (WHO) reported that around 9 million people died from cancer in 2016, positioning it as the second most common cause of death globally after cardiovascular diseases.^1^ The same trend holds true in Germany, where cancer is also ranked second in terms of mortality and was responsible for the death of more than 220,000 individuals in 2015.^2^ Moreover, cancer treatments require long‐term, intensive use of healthcare services, commonly bear adverse health effects, and may limit the ability of patients to work. These potential losses in earnings together with substantial raises in healthcare expenditures cause many cancer patients to become subject to financial vulnerability.^3^, ^4^, ^5^, ^6^

Cancer patients might experience financial hardship primarily as a result of increasing out of pocket (OOP) expenses and reduced working hours and productivity after their diagnosis. Numerous studies have explored this issue; most of them originating from the United States, to a lesser extent from Europe, where evidence from Germany is limited.^3^, ^7^, ^8^, ^9^, ^10^, ^11^ This geographical bias likely responds to financial stress associated with cancer treatment being less pronounced under healthcare systems with uniform coverage and capped co‐payments,^12^ leading to the perception that financial vulnerability is non‐existent for cancer patients when access to healthcare is universal. Contrary to this expectation, the few existing studies reveal that financial hardship is not uncommon among cancer patients in Germany.^13^, ^14^, ^15^, ^16^, ^17^, ^18^, ^19^ These are mostly based on single hospital surveys, and to our knowledge, there is not currently any research performed at the national level.

Certainly, the availability and extent of the German healthcare and social security systems defray a large proportion of the costs to patients for cancer treatment. Health insurance coverage encompasses virtually the entire population and benefits are generous in both scope and scale, constraining the spending amount incurred in healthcare for cancer patients.^20^, ^21^ Government officials and experts from oncology organizations usually agree that drugs for common cancer types are consistently available and are fully reimbursed in Germany.^22^, ^23^ In addition, social security schemes are offered in forms of paid sick leave, unemployment benefits and early pension in order to offset income losses derived from temporary or permanent work leave.^24^

Nevertheless, existing social safety nets do not entirely protect cancer patients from financial vulnerability in Germany. For example, the Law for the Modernization of the Statutory Health Insurance (SHI) in 2004 led to a considerable increment in patient's contributions in medical treatment, medicine and transportation costs.^24^ OOP costs amount to around 10% for outpatient prescriptions, physiotherapy and visits to the doctor within the SHI. A fixed fee is charged per hospitalization day, and travel costs for outpatient treatment are usually not covered.^25^ Furthermore, social security programs often just partially compensate income losses or guarantee only a minimum subsistence in case of work inability. Employers are required to pay full salary for sick leave up to six weeks, after which the SHI may compensate on average 70% of gross income via a sick pay for a maximum period of 78 weeks.^24^ After the sick pay period has ended, cancer patients can either apply for the unemployment benefit II or a disability pension, equivalent to an amount of 400 euros per month plus payment of rent and up to 50% of the net income, respectively.^24^, ^25^ Some of these benefits may be collected only by individuals fulfilling certain requirements.^24^, ^25^

Recent surveys indicate that many cancer patients still experience large expenditure increases and income losses in Germany. These studies reported substantial OOP payments associated with hospital stays, transportation and medication, as well as diminishing working time and significant reductions in income. For instance, surveys administrated to cancer patients in hospital settings suggest that a proportion between a third and a half of the interviewed did not return to work after cancer treatment.^26^, ^27^, ^28^, ^29^ Dietsche^16^ analyzed routine data of a SHI and obtained similar results. Other studies identified that, although a large proportion failed to return to work in the short‐run, a share between 70% and 87% were back to work after a one‐year absence.^30^, ^31^, ^32^ An earlier survey by Bikowski^14^ examined 154 cancer patients at the National Center for Tumor Disease (NCT) in Heidelberg and showed drops in monthly income from work to be between 100 and 500 euros for more than 60% of those surveyed, and greater than 1200 euros for 12%. A series of studies involving 247 advanced stage patients with colorectal and neuroendocrine tumors performed also at the NCT in Heidelberg found that around a third of the sample patients stated a significant drop in net household income after diagnosis.^12^, ^13^, ^17^, ^18^, ^19^ In two of these studies, patients who reported a net household income loss indicated this amount to be at least 800 euros per month in 44% and 45% of the cases, respectively.^12^, ^13^ Moreover, one of these studies revealed that income losses outweigh OOP costs. While monthly OOP payments did not exceed 200 euros in 77% of affected patients, 24% of those with income losses stated these to be more than 1200 euros per month.^12^ Büttner, König^15^ estimated OOP costs also to be moderate with an average between 200 and 150 euros per quarter year in a sample of 502 cancer patients from 16 clinics in Leipzig. Other surveys focusing on quality of life of cancer patients found that financial security is one of the areas with the least satisfaction.^33^, ^34^ Bikowski ^14^ also observed that a considerable proportion of cancer patients are either under sick pay or disability pension (20% and 6%, respectively).

This evidence suggests that cancer patients may still face healthcare expenditures and, most notably, large income losses after their diagnosis, despite nearly full health insurance coverage of anti‐cancer treatments and medications as well as extensive social security programs in Germany.^35^ By making use of one of the largest and most comprehensive household surveys in Germany, the present study seeks to provide evidence on the magnitude of the income loss side of financial hardship and to overcome shortcomings in previous research. Existing literature on the subject is founded in single cancer center samples or restricted to particular geographical regions, and hence their conclusions are limited in scope and difficult to generalize. Our sample covers a large number of individuals at the national level with a wide range of individual and household characteristics restricting biases derived from respondent under‐representation in small samples. Likewise, having numerous personal characteristics captured with this survey allows the analysis to disentangle the effect of a cancer diagnosis from that of other income drivers, which is difficult to achieve when information on only a constrained number of co‐founders is available. In addition, as the sample includes healthy individuals, we can observe the impact that is attributable to cancer, unlike with surveys targeted to cancer patients only. These features of our sample should result in a more precise estimation of the magnitude of income loss after a cancer diagnosis in Germany.

## 2 METHODS

This study analyzes data from the Socio Economic Panel (SOEP) household survey implemented by the German Institute of Economic Research (DIW). It is a longitudinal panel, which started in 1984, and interviews adult household members annually. It is the largest and most comprehensive household survey in Germany, consisting of around 20,000 individuals from 12,000 households.^36^ Beginning in 2009, individuals aged 16 years or older and who are in the labor force are asked if they have been ever diagnosed with any of nine common diseases, including cancer. This question is asked every 2 years, and at the time of being granted access to the survey, there were four different time points available for this item, namely 2009, 2011, 2013, and 2015. The overall response rate for this question is of 75% for the whole period. The numbers of individuals self‐reporting a cancer diagnosis are 792, 956, 1057, and 1184, respectively, corresponding to 3.8%, 4.5%, 5.5%, and 4.7% of those answering the question. Although individuals are followed over time, the panel is unbalanced as new individuals enter the sample in each wave, while others might leave it as a result of death or other reasons.

Two different sample consistency checks were performed to guarantee that only reliable cases were included in the analysis. The first of these tests ensured period consistency, meaning that an individual selecting any disease diagnosis and the no disease diagnosis option simultaneously for any given year is considered an inconsistent observation. Such cases were very rare: in the sample, on average, only four of those cases per year were identified. Given the wording of the question, the second check ensured time consistency by certifying that an individual who reports a cancer diagnosis in a given year also reports a cancer diagnosis in every subsequent year. Moreover, because the sample contains missing values (e.g., when an individual is not interviewed in one of the four years) a strict time consistency check, which excludes series with incomplete information, was conducted in addition to the aforementioned check. The final sample fulfilled the period and time consistency checks, and the strict time consistency check was further applied to test for the robustness of results. The percentage of observations that follows the time consistency condition is higher in 2009 than for the other years, as the absence of a previous period makes the condition less likely to be rejected. The empirical strategy addresses this bias with the inclusion of time year dummy variables.

The analysis focused on four different outcomes: job income, work status, working hours, and pensions. Job income constitutes the sum of salary and wages from the main job, income from secondary employment and income from self‐employment for the individual in a given year; it does not comprise social benefits or other transfer payments. Work status options include full‐time, part‐time, or unemployment, while working hours refer to annual work hours of the individual in a given year. Pensions includes old‐age, disability and civil servant pensions, widow and orphan pensions, company pension and private pension for the individual in a given year. Except for work status, all of these items in the survey are open‐ended questions. A single analysis of each of the elements which compose the aggregates for job income and pensions was not feasible, as some of them are limited in the number of observations and might lead to inefficient estimators. Sample averages were calculated for these four outcomes and by cancer diagnosis to identify any patterns. This initial evidence was further explored with an empirical strategy that models the four different outcomes as a function of a cancer diagnosis. A first regression equation is as follows:

(1) $$\begin{array}{cc} {\text{Outcome}}_{\mathrm{it}} & =\beta_{0}+\beta_{1}*{\text{CDiag}}_{\mathrm{it}}+\beta_{2}*{\text{Comorb}}_{\mathrm{it}}+\beta_{3}*{\text{Gender}}_{\mathrm{it}}\times \beta_{4}*{\text{HHMem}}_{\mathrm{it}}\\ & +\beta_{5}*{\text{Age}}_{\mathrm{it}}+\beta_{6}*{\text{Age}}_{\mathrm{it}}^{2}+\beta_{7}*{\text{Edu}}_{\mathrm{it}}+\beta_{8}*{\text{Working}}_{\mathrm{it}}+\mu_{i}+\tau_{t}+\varepsilon_{\mathrm{it}} \end{array}$$

Outcome*_it_* is the outcome variable to be estimated of individual *i* in year *t*. Except for the work status, which is a categorical variable, the outcome variables are all in logarithmic form. CDiag*_it_* is the main explanatory variable signaling the cancer diagnosis status. In a first version, it is a dummy variable that takes the value of 0 if individual *i* reports no cancer diagnosis in year *t* (labeled as “no cancer diagnosis”) and 1 otherwise (labeled as “cancer diagnosis”). In a second version, it takes the value of 0 if individual *i* reports no cancer diagnosis in year *t* and in any other year (labeled as “non‐cancer control”), the value of 1 if individual *i* reports no cancer diagnosis in year *t* but reports a cancer diagnosis in any other year (labeled as “before cancer diagnosis”), and the value of 2 if individual *i* reports a cancer diagnosis in year *t* (labeled as “cancer diagnosis”). Comorb*_it_*, Gender*_i_*, HHMem*_it_*, Age*_it_*, Edu*_it_*, and Working*_it_* control for others characteristics of the individuals, namely the number of comorbidities, gender, household position, age, education level and working status of individual *i* in year *t*, respectively. Comorb_it_ is a six‐level categorical variable that is introduced in the regression equation as dummy variables signaling each category the variable might take: 0 comorbidities, 1 comorbidity, 2 comorbidities, 3 comorbidities, 4 comorbidities and 5 comorbidities. Gender*_i_* is a dummy variable that assigns a value of 1 if the respondent is a woman and 0 otherwise. HHMem*_it_* is a five‐level categorical variable transformed into a set of dummy variables in the regression equation indicating each category: household head, partner, child, relative and non‐relative. Age*_it_* is a continuous variable and Age^2^ *_it_* its squared form allowing for a non‐linear relationship between the age of the respondent and the outcome. Edu*_it_* is a three‐level categorical variable addressed in the regression equation by dummy variables for each category: less than high school, high school and more than high school. Finally, Working*_it_* is a dummy variable that takes the value of 1 if the respondent is currently working and 0 otherwise. The first level of the categorical variables is assumed as baseline category. The construction of these control variables is found in detailed in Appendix 1. State of residence and year fixed effects are denoted by μ*_i_* and τ*_t_*, and ε*_it_* is the error term. Lastly, β coefficients in the models with continuous outcome variables were estimated with an ordinary least squares (OLS) estimator, likewise, a multinomial logistic estimator for the model with the categorical outcome variable.

The second regression equation below was executed across a sample containing only individuals that are actively working. Outcome*_it_* is thus in this equation conditional to individual *i* being actively working in year *t*. In this way, it could be observed if the effect of a cancer diagnosis on the outcomes held when censoring the unemployed population. The outcome variables, work status and pension, could not be introduced in this model, as variation is limited. While cases of individuals actively working and receiving a pension are rare, the working status outcome loses a category when restricted to the working population sample. For this reason, the control variable Working*_it_* was also omitted, and a working sector fixed effects variable, denoted by ω*_i_*,_,_ was included. Outcome variables are all in logarithmic form and β coefficients were to be estimated by means of an OLS model.

(2) $$\begin{array}{cc} {\text{Outcome}}_{\mathrm{it}} & =\beta_{0}+\beta_{1}*\text{CDiag}+\beta_{2}*\text{Comorb}+\beta_{3}*\text{Gender}+\beta_{4}*\text{HHMem}+\beta_{5}*\text{Age}+\beta_{6}*{\text{Age}}^{2}\\ & +\beta_{7}*\text{Edu}+\omega_{i}+\mu_{i}+\tau_{t}+\varepsilon_{\mathrm{it}} \end{array}$$

A detailed description of the variables included in the models is found in Appendix 1, and summary statistics for these variables in Appendix 2. Appendix 3 presents correlations between control variables for the whole sample and the working population sample, employed to estimate Equation (1) and Equation (2), respectively. Output results for Equation (1) and Equation (2) are presented in Table 1 and Table 2, respectively. Estimated coefficients are considered statistically significant at conventional levels if a *p*‐value of at least 10 percent is reached. *p*‐value levels of at least 10 percent are denoted in the tables with an asterisk (*).

**TABLE 1:** Effect of Cancer Diagnosis on Different Outcomes for the Whole Sample in 2009–2015

	Work status						
	Job income (1)	Job income (2)	Part time (3)	Not working (4)	Working hours (5)	Pension (6)	Pension (7)
Before cancer diagnosis		−0.123					
		0.075					
Cancer diagnosis	−0.300*	−0.334*	0.082	0.458*	−0.269*	0.021	0.016
	0.051	0.055	0.069	0.068	0.044	0.014	0.014
1 comorbidity	−0.121*	−0.121*	0.070*	0.214*	−0.090*	−0.001	−0.009
	0.027	0.027	0.025	0.028	0.023	0.018	0.019
2 comorbidities	−0.355*	−0.353*	0.175*	0.597*	−0.291*	−0.016	−0.025
	0.038	0.038	0.039	0.04	0.032	0.019	0.02
3 comorbidities	−0.658*	−0.656*	0.513*	1.360*	−0.524*	0.001	−0.01
	0.055	0.055	0.069	0.063	0.047	0.024	0.026
4 comorbidities	−0.609*	−0.604*	1.171*	2.023*	−0.499*	−0.034	−0.069
	0.114	0.114	0.178	0.165	0.099	0.046	0.049
5 comorbidities	−1.759*	−1.761*	1.237	4.589*	−1.390*	−0.209*	−0.191
	0.306	0.306	1.008	0.737	0.264	0.113	0.129
Gender	−0.664*	−0.663*	1.793*	1.520*	−0.441*	−0.343*	−0.367*
	0.022	0.022	0.022	0.025	0.019	0.015	0.015
Partner	−0.119*	−0.119*	0.230*	0.286*	−0.115*	−0.390*	−0.456*
	0.023	0.023	0.022	0.026	0.02	0.013	0.013
Child	−0.452*	−0.451*	0.274*	0.439*	−1.094*	0.05	−0.206
	0.045	0.045	0.049	0.051	0.038	0.069	0.173
Relative	−0.239*	−0.240*	0.388*	0.544*	−0.280*	−0.132*	−0.094
	0.128	0.128	0.173	0.175	0.11	0.059	0.059
Non‐relative	−0.174	−0.175	0.502*	0.428*	−0.446*	−0.435*	−0.629*
	0.155	0.155	0.185	0.197	0.133	0.107	0.144
Age	0.161*	0.161*	−0.264*	−0.596*	0.178*	0.067*	0.077*
	0.004	0.004	0.006	0.006	0.003	0.004	0.011
Age^2^	−0.002*	−0.002*	0.003*	0.007*	−0.002*	−0.000*	−0.000*
	0.000	0.000	0.000	0.000	0.000	0.000	0.000
High school	0.616*	0.616*	−0.386*	−1.105*	0.729*	0.240*	0.236*
	0.03	0.03	0.034	0.035	0.026	0.018	0.019
More than high school	1.090*	1.091*	−0.723*	−1.733*	0.903*	0.648*	0.686*
	0.035	0.035	0.038	0.041	0.03	0.023	0.024
Working	6.138*	6.137*			4.136*	−0.175*	−0.090*
	0.022	0.022			0.019	0.012	0.015
Constant	0.426*	0.423*	4.188*	10.809*	−1.182*	6.585*	6.273*
	0.109	0.109	0.146	0.153	0.094	0.128	0.424
State fixed effects	Yes	Yes	Yes	Yes	Yes	Yes	
Year fixed effects	Yes	Yes	Yes	Yes	Yes	Yes	
Sector fixed effects	No	No	No		No	No	No
*N*	81,232	81,232	81,234		81,232	23,640	19,240
r2	0.78	0.78	—		0.71	0.32	0.33

Notes

The unit of analysis is individual *i* in year *t*. Column labels denote the dependent variable employed in the respective model specification. The dependent variable is job income in columns (1) and (2), work status in columns (3) and (4), working hours in column (5) and pension in columns (6) and (7). Results for the working status outcome are presented relative to the full‐time category: for the “part‐time” category in column (3) and for the “not working” category in column (4). Sample is restricted to individuals aged 65 and older in column (7). Estimated coefficients of the variable are reported, standard errors are shown below the coefficients.

* *p* < 0.1.

**TABLE 2:** Effect of Cancer Diagnosis on Different Outcomes for the Working Population Sample in 2009–2015

	Job income (1)	Job income (2)	Working hours (3)
Before cancer diagnosis		0.096	
		0.077	
Cancer diagnosis	−0.204*	−0.159*	−0.145*
	0.053	0.064	0.062
1 comorbidity	−0.077*	−0.078*	−0.045*
	0.024	0.024	0.025
2 comorbidities	−0.131*	−0.133*	−0.063
	0.041	0.041	0.043
3 comorbidities	−0.380*	−0.381*	−0.240*
	0.075	0.075	0.078
4 comorbidities	−0.516*	−0.528*	−0.316
	0.206	0.207	0.214
5 comorbidities	−0.957	−0.954	−0.028
	1.097	1.097	1.191
Gender	−0.675*	−0.676*	−0.463*
	0.021	0.021	0.022
Partner	−0.078*	−0.078*	−0.066*
	0.02	0.02	0.022
Child	−0.181*	−0.181*	−0.831*
	0.035	0.035	0.04
Relative	−0.11	−0.11	−0.224*
	0.119	0.119	0.133
Non‐relative	−0.349*	−0.349*	−0.545*
	0.123	0.123	0.143
Age	0.177*	0.177*	0.332*
	0.005	0.005	0.005
Age^2^	−0.002*	−0.002*	−0.004*
	0.000	0.000	0.000
High school	0.473*	0.473*	1.013*
	0.029	0.029	0.031
More than high school	0.900*	0.900*	1.104*
	0.033	0.034	0.036
Constant	5.519*	5.520*	−0.975*
	0.126	0.126	0.14
State fixed effects	Yes	Yes	Yes
Year fixed effects	Yes	Yes	Yes
Sector fixed effects	Yes	Yes	Yes
*N*	47,499	47,499	47,499
r2	0.2	0.2	0.25

Notes

The unit of analysis is individual *i* in year *t*, conditional to individual *i* being actively working in year *t*. Column labels denote the dependent variable employed in the respective model specification. The dependent variable is job income in columns (1) and (2) and working hours in column (3). Estimated coefficients of the variable are reported, standard errors are shown below the coefficients.

* *p* < 0.1.

## 3 RESULTS

**FIGURE 1:**
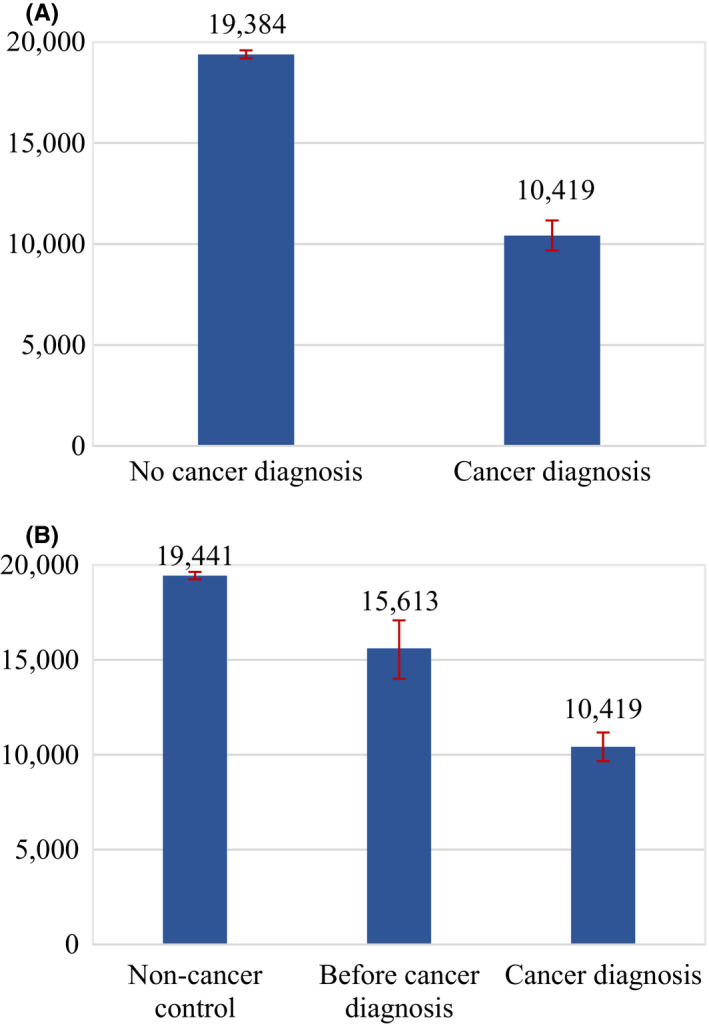
(A) Job Income Average in Constant Euro by Cancer Diagnosis Status (Two Categories) for the Whole Sample in 2009–2015. Note: Job income average in constant 2016 euro per individual and year for the period 2009–2015. “No cancer diagnosis” denotes observations in which no cancer diagnosis is reported, and “Cancer diagnosis” denotes observations in which a cancer diagnosis is reported. Interval bars denote 95% confidence intervals. (B). Job Income Average in Constant Euro by Cancer Diagnosis Status (Three Categories) for the Whole Sample in 2009–2015. Note: Job income average in constant 2016 euro per individual and year for the period 2009–2015. “Non‐cancer control” denotes observations in which no cancer diagnosis is reported from those individuals that never report a cancer diagnosis at any other point the sample, “Before cancer diagnosis” denotes observations in which no cancer diagnosis is reported from those individuals that do report a cancer diagnosis at any other point in the sample, and “Cancer diagnosis” denotes observations in which a cancer diagnosis is reported. Interval bars denote 95% confidence intervals

**FIGURE 2:**
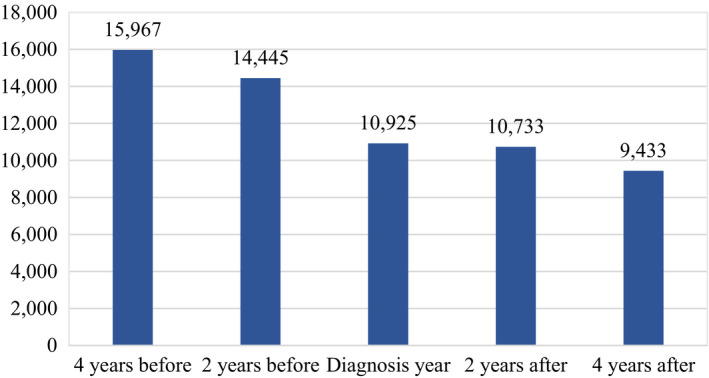
Job Income Average in Constant Euro by Reference Year for the Cancer Patient Sample in 2009–2015. Note: Job income average in constant 2016 euro per individual reporting a cancer diagnosis and by reference year. “Diagnosis year” denotes observations in the year for which the cancer diagnosis was reported, “4 years before” denotes observations in the year four years immediately before the cancer diagnosis was reported, “2 years before” two years immediately before, “2 years after” two years immediately after and “4 years after” four years immediately after

**FIGURE 3:**
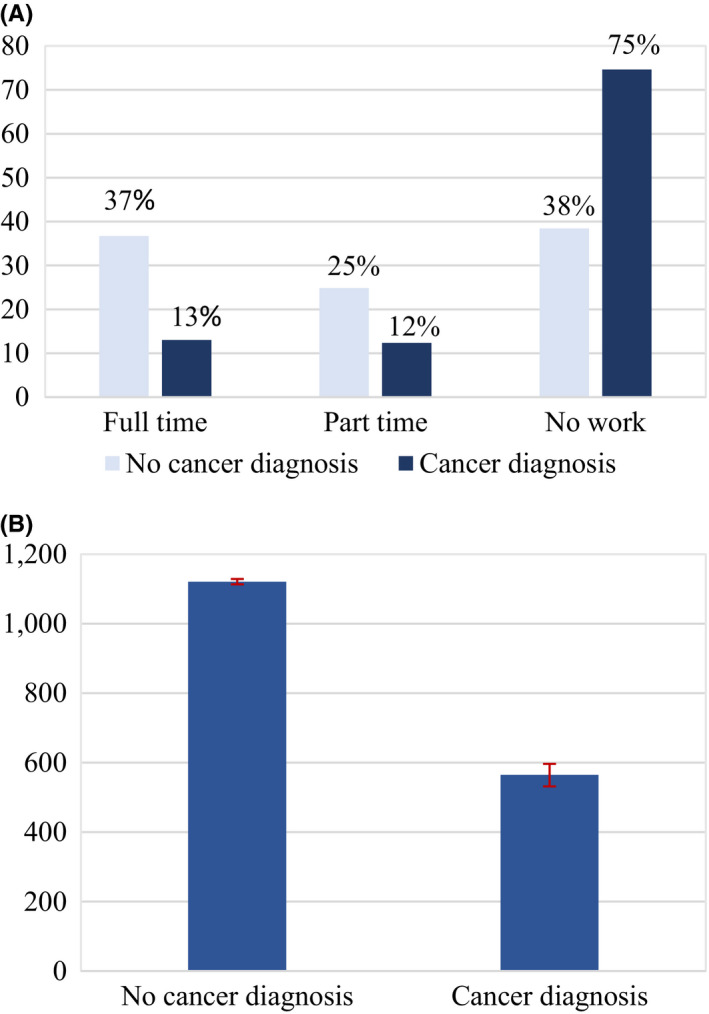
(A) Percentage of Individuals by Work Status and Cancer Diagnosis for the Whole Sample in 2009–2015. Note: Percentage of individuals in each work status category for the period 2009–2015. “No cancer diagnosis” denotes observations in which no cancer diagnosis was reported, and “Cancer diagnosis” denotes observations in which a cancer diagnosis was reported. (B). Working Hours Average by Cancer Diagnosis for the Whole Sample in 2009–2015. Note: Working hours average per individual and per year for the period 2009–2015. “No cancer diagnosis” denotes observations in which no cancer diagnosis was reported, and “Cancer diagnosis” denotes observations in which a cancer diagnosis was reported. Interval bars denote 95% confidence intervals

**FIGURE 4:**
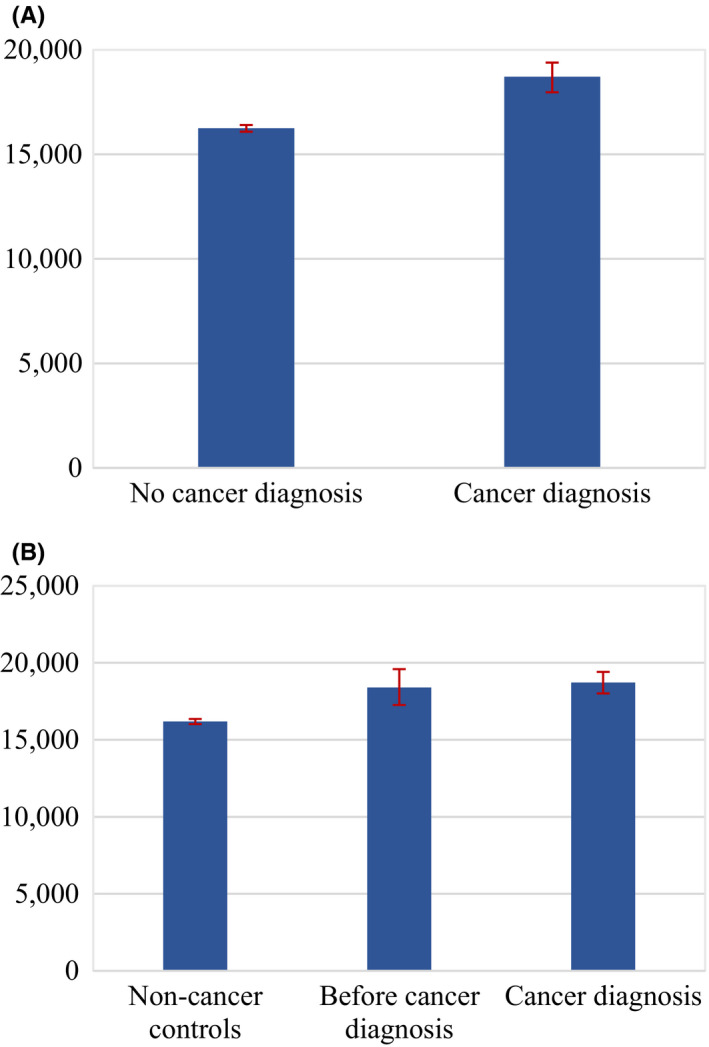
(A) Pension Average in Constant Euro by Cancer Diagnosis Status (Two Categories) for the Whole Sample in 2009–2015. Note: Pension average per individual and year for the period 2009–2015. “No cancer diagnosis” denotes observations in which no cancer diagnosis is reported, and “Cancer diagnosis” denotes observations in which a cancer diagnosis is reported. Interval bars denote 95% confidence intervals. (B) Pension Average in Constant Euro by Cancer Diagnosis Status (Three Categories) for the Whole Sample in 2009–2015. Note: Pension average per individual and year for the period 2009–2015. “Non‐cancer control” denotes observations in which no cancer diagnosis is reported from those individuals that never report a cancer diagnosis at any other point the sample, “Before cancer diagnosis” denotes observations in which no cancer diagnosis is reported from those individuals that do report a cancer diagnosis at any other point in the sample, and “Cancer diagnosis” denotes observations in which a cancer diagnosis is reported. Interval bars denote 95% confidence intervals

Initial evidence was captured with sample averages for the four different outcomes and distinguishing by the cancer status of individuals. Job incomes were considerably lower for periods in which a cancer diagnosis is reported, as observed in Figure 1A and 1B. They were on average 10,419 euros when a cancer diagnosis is reported and 19,384 euros when it is not. In addition, job incomes remained substantially lower two and four years after the diagnosis is initially reported, as shown in Figure 2. Similarly, full‐time work was more common among individuals reporting no cancer diagnosis, translating as well into more working hours, as depicted in Figure 3A and 3B. While 13% of individuals that reported a cancer diagnosis work full‐time, 37% of individuals that did not report a cancer diagnosis do so. On the contrary, average pension levels per individual did not seem to differ substantially between those reporting and not reporting a cancer diagnosis, as seen in Figure 4A and 4B. These were 18,715 euros for periods in which a cancer diagnosis is reported and 16,247 euros in those in which it is not.

Regression results in Table 1 present the effect of reporting a cancer diagnosis in the four outcomes: job income, work status, working hours and pensions. Column headers denote the outcome variable in the respective model specification. Cancer diagnoses were associated with lower job incomes. As seen in columns 1 and 2, the reduction in job incomes was statistically significant within the year the cancer diagnosis is reported. The drop rate was between 28% and 26% depending on main explanatory variable employed. Percentage changes were obtained by transforming the corresponding coefficient with exp(*β_x_*)—1. Relative to working full‐time, reporting a cancer diagnosis did not significantly increase the likelihood of working part‐time, as observed in column 3. It did increase, however, the likelihood of not working significantly, as observed in column 4. The decrease in the number of working hours as a result of a cancer diagnosis was also statistically significant and of 24% in size, as exhibited in column 5. On the other hand, the level of pensions did not seem to be significantly affected after reporting a cancer diagnosis. This result held when the sample was restricted to the population 65 years of age and older, as shown in columns 6 and 7.

Table 2 exhibits regression results measuring the influence of reporting a cancer diagnosis in the outcomes job income and working hours across the working population sample. As before, column headers refer to the outcome variable in the respective model specification. The impact of a cancer diagnosis on job income remained negative and statistically significant, as observed in columns 1 and 2. The drop rate is between 15% and 18%, depending on the main explanatory variable employed. Similarly, the number of working hours was significantly reduced as a result of a cancer diagnosis and at a rate of 13%. It is noteworthy that r‐square values were lower for these regressions compared to those founded for the entire sample.

The analysis presented in Table 3 explores the persistence of the job income effect after a cancer diagnosis is reported. For this purpose, time lags for the cancer diagnosis variable were incorporated. Only individuals who report a cancer diagnosis in any year are taken into account because time lags require a variation over time in order to be estimated. For this reason, the sample size is considerably smaller. In addition, a longer time lag resulted in a smaller sample size, given that a point in time is lost with each lag. As the panel is defined every two years, the first time lag corresponds to a two‐year variable lag and the second time lag to a four‐year variable lag. Note that the effect of the cancer diagnosis in *t*‐*x* on the outcome in *t* is equivalent to the effect of the cancer diagnosis in *t* on the outcome variable in *t*+*x*. For this reason, we interpret the coefficient of the lagged variable in *x* periods, as the effect of a cancer diagnosis on the outcome *x* periods after the diagnosis. The contemporaneous impact of reporting a cancer diagnosis was negative and statistically significant, as exhibited in column 1. The size of the effect was similar to those previously estimated. The coefficient for the first time lag of the cancer diagnosis variable was negative as well and statically significant, being smaller in size in comparison to the contemporaneous effect, as seen in column 2. Conversely, the coefficient for the second time lag, shown in column 3, was not statically significant.

**TABLE 3:** Lagged Effect of Cancer Diagnosis on Different Outcomes for the Cancer Patient Sample in 2009–2015

	Job income (1)	Job income (2)	Job income (3)
Cancer diagnosis	−0.242*		
	0.085		
Cancer diagnosis t‐2		−0.177*	
		0.093	
Cancer diagnosis t‐4			−0.077
			0.117
1 comorbidity	−0.169	−0.109	0.107
	0.109	0.130	0.155
2 comorbidities	−0.294*	−0.265*	−0.319*
	0.126	0.148	0.176
3 comorbidities	−0.635*	−0.530*	−0.392*
	0.158	0.184	0.219
4 comorbidities	−0.331	−0.295	−0.408
	0.273	0.311	0.370
5 comorbidities	−1.025	−0.434	
	1.351	2.144	
Gender	−0.460*	−0.410*	−0.419*
	0.093	0.110	0.130
Partner	−0.133	−0.183	−0.092
	0.094	0.114	0.136
Child	−0.707	−0.858	−0.589
	0.436	0.630	0.953
Relative	0.385	0.230	−1.268
	0.803	1.036	2.111
Non−relative	−1.252	−0.778	−1.000
	1.025	1.287	1.705
Age	0.021	0.024	0.007
	0.023	0.031	0.040
Age^2^	−0.001*	−0.001*	−0.000
	0.000	0.000	0.000
High school	0.438*	0.420*	0.303
	0.137	0.171	0.214
More than high school	0.739*	0.694*	0.500*
	0.155	0.190	0.234
Working	7.419*	7.591*	7.991*
	0.099	0.127	0.160
Constant	3.294*	2.782*	2.795*
	0.742	1.030	1.319
State fixed effects	Yes	Yes	Yes
Year fixed effects	Yes	Yes	Yes
Sector fixed effects	No	No	No
*N*	4156	2692	1502
r2	0.813	0.814	0.823

Notes

The unit of analysis is individual *i* in year *t*, conditional to individual *i* reporting a cancer diagnosis in any year *t*. Column labels denote the dependent variable employed in the respective model specification. The dependent variable is job income in columns (1) to (3). Estimated coefficients of the variable are reported, standard errors are shown below the coefficients.

* *p* < 0.1.

Finally, in a first robustness test, the effects for men and women were analyzed separately. An interaction term between the CDiag and Gender variables was introduced in the regression equations for the four outcomes and executed among the full and the working population samples. Nonetheless, the interaction term usually failed to be statistically significant across all models specifications, therefore we cannot conclude that the effect on the various outcomes is different for men and women. In a second robustness test, regression coefficients were re‐estimated with the sample that fulfills the strict time consistency check, which excludes observations with incomplete information. Estimations remained stable in terms of sign and significance levels. These results are available upon request.

## 4 DISCUSSION

The present study provides evidence for a topic that is poorly understood Germany. The fact that anti‐cancer treatments and medications are commonly accessible and the extent of social security is ample, unlike in some other countries in Europe, reinforces the belief that financial hardship is not a major concern for cancer patients in Germany.^22^, ^23^ Contrary to this view, some previous studies based on hospital surveys suggest that cancer patients do face important OOP expenses and, mostly notably, large losses in income in Germany.^13^, ^14^, ^15^, ^16^, ^17^, ^18^, ^19^ This study provides evidence on the magnitude of absolute income loss at a national level and overcomes shortcomings in previous literature to more precisely measure the impact of a cancer diagnosis. It focuses on changes in job income, work status, working hours and pensions; point estimates were obtained for each of the single effects as well as for their persistence over time.

Results showed that job incomes decrease between 26% and 28% within the year a cancer diagnosis was reported. The study also found that reporting a cancer diagnosis increased the likelihood of work inactivity and reduced the number of working hours by 24%. Lower levels in job income and working hours were also encountered when the analysis was restricted to the working population only. The effect in income persisted two years after the cancer diagnosis was reported, but was not observable four years thereafter. This result might be a consequence of differences in patient characteristics that are not addressed by the control variables. Patients that leave the sample quickly are more likely to be in a late cancer stage and face larger income loses. On the other hand, patients that remain in the sample over a long period of time are more likely to be in an early cancer stage and therefore in a healthier status that allow them to return to their job. For example, cancer patients that left the sample two years after a reported cancer diagnosis experienced a drop in income per year of 28% on average, while the same figure is of 18% for those leaving the sample four years after the cancer diagnosis is reported. In contrast, pension levels were not significantly affected by a cancer diagnosis. This may be due to those within the population who already receive a pension having been entitled to it before a cancer diagnosis. Importantly, this evidence illustrates that cancer patients are more exposed to financial hardship when the cancer diagnosis occurs before requirements to obtain a pension are met.

The absolute fall in job income predicted by the empirical strategy in this study cannot be directly compared with the findings from previous studies because it consists of a point estimate while existing research provides information on the frequency of predefined income intervals. Nevertheless, amount sizes are relatable. With an average job income of 15,613 euros prior to reporting a cancer diagnosis, the econometric models in this study forecasted an absolute fall in job income per patient between 4059 euros and 4371 euros per year, or between 338 euros and 364 euros per month, during those years in which a cancer diagnosis was reported. Corresponding numbers were between 100 to 500 euros per month for 60% of cancer patients in Bikowski,^14^ and below 800 euros per month for 87% of cancer patients in Apostolidis, Mehlis ^13^ and for 83% of cancer patients in Mehlis, Witte.^12^ Our estimation, however, disentangles the effect of a cancer diagnosis from that of comorbidities, gender, household position, age, education level, and working status, whereas results from the previous research do not. We can, therefore, be more confident that the predicted changes in job incomes are, to greater extent, associated to a cancer diagnosis.

Other countries being evaluated on the impact on income and founded on longitudinal national registries or household surveys is observed for Denmark,^37^, ^38^ Norway,^39^, ^40^ Sweden ^41^ and the United States.^42^ Additional articles based on national wide surveys to cancer patients include Canada ^43^ and the Netherlands.^44^ Except for Eaker, Wigertz,^41^ all the studies identify a significant drop in income, varying from 3% to 40% within the first year after the cancer diagnosis. Figures close to the lower limit are found in the Scandinavian countries, while those close to the upper limit in Canada and the United States. Our estimates fall within the latter group. The discrepancy in the results from the Scandinavian studies is very likely a consequence of the income definition they employ. They specify income as job earnings plus social benefits and other transfer payments.^37^, ^38^, ^39^, ^40^, ^41^ This means their analyses also address income compensation schemes in case of work incapacity, which in turn lessen the adverse outcomes. On the other hand, the income definition in our study, as well as in Hopkins, Goeree,^43^ and Zajacova, Dowd^42^ for Canada and the United States, respectively, refers merely to job earnings. In addition, some of the studies for the Scandinavian countries comprise breast cancer patients only, including Eaker, Wigertz,^41^ who usually present relatively better prognosis compared to other cancer types.^37^, ^38^ Moreover, the rebound income effect that we obtained four years after the cancer diagnoses, is also identified in Zajacova, Dowd^42^ after 4 years for family income, and in Jensen, Overgaard^38^ after seven years for personal income. Zajacova, Dowd^42^ categorizes the rebound in income as relative to the pre‐diagnosis income, equally as in our study, while Jensen, Overgaard^38^ relative to the control group income.

A major limitation of this study is the self‐reported nature of the cancer diagnosis variable, which is subject to measurement error. Although the survey does not verify confirmed cancer cases, the implementation of period and time consistency checks decreases the likelihood of false positive observations appearing in the sample. This way, biases due to measurement error are limited. In addition, the coefficient measuring the impact of a cancer diagnosis is robust to different identification strategies and sample sizes. Another limitation of this study is the omission of the healthcare expenditure side of financial hardship in the analysis. The SOEP survey does not record OOP costs and therefore these cannot be measured. Research in this topic at a national level is still missing. Lastly, this study cannot address differences across cancer sites or any other epidemiological characteristics. The SOEP survey does not provide such information which has been identified to affect the size of the financial burden.^45^, ^46^

## 5 CONCLUSION

This study measured the extent of the income loss component in financial hardship from cancer patients in Germany with a nationwide survey. In particular, it examined changes in job income, work status, working hours and pension as a result of a cancer diagnosis. Our results show that job incomes drop between 26% and 28% within the year a cancer diagnosis was reported. This effect persisted for two years after the diagnosis and vanished four years thereafter. Furthermore, analyses revealed increases in the likelihood of unemployment and the reduction of working hours after a cancer diagnosis. However, pension levels are not affected by a cancer diagnosis. This suggests that the exposure to financial hardship is more critical when the cancer diagnosis occurs during the active work life and before requirements to obtain a pension are met. Current social security schemes protect cancer patients of certain work backgrounds only, and when they do, they offset income partially and only for a limited period of time. Self‐employed workers, students, and persons insured by their families are particularly at risk, as well as anyone with a work incapacity for a duration longer than one and a half years. This set of circumstances still needs to be acknowledged by the authorities and be called to the attention of policy makers to design a more inclusive and prolonged compensation mechanism to prevent cancer patients and their families from reaching poverty as well as to identify vulnerable groups.

## ETHICS STATEMENT

Given the nature of secondary data analysis, the need for an ethics approval was waived. The study was performed in accordance with the Declaration of Helsinki and follows the principles of Good Practice in Secondary Data Analysis.

## CONFLICT OF INTEREST

The authors certify that they have no affiliations with or involvement in any organization or entity with any financial interest (such as honoraria; educational grants; participation in speakers’ bureaus; membership, employment, consultancies, stock ownership, or other equity interest; and expert testimony or patent‐licensing arrangements), or non‐financial interest (such as personal or professional relationships, affiliations, knowledge or beliefs) in the subject matter or materials discussed in this manuscript.

## Data Availability

The data that support the findings of this study are available on request from the corresponding author. The data are not publicly available due to privacy or ethical restrictions.

## APPENDIX 1

### Variables Definitions and Sources

**Table:**

Variable	Definition	Source
Job income	Sum of salary and wages from main job, income from secondary employment and income from self‐employment for the individual in a given year.	SOEP ^47^
Work status	Categorical variable that signals the work status of the individual in a given year. It is coded 1 if working full‐time, 2 if working part‐time and 3 if unemployed.	SOEP ^47^
Working hours	Annual work hours of the individual for a given year.	SOEP ^47^
Pension	Sum of old‐age, disability and civil servant pensions, widow and orphan pensions, company pension and private pensions for the individual in a given year.	SOEP ^47^
Non‐cancer control	Baseline category. Dummy variable coded 1 if the individual reports no cancer diagnosis in a given year and in any other year, and 0 otherwise. Respectively, “Cancer” option is omitted in the disease diagnosis question, as well as in any other year.	SOEP ^47^
Before cancer diagnosis	Dummy variable coded 1 if the individual reports no cancer diagnosis in a given year but reports a cancer diagnosis in any other year, and 0 otherwise. Respectively, “Cancer” option is omitted in the disease diagnosis question, but selected in any other year.	SOEP ^47^
Cancer diagnosis	Dummy variables coded 1 if the individual reports a cancer diagnosis in a given year, and 0 otherwise. Respectively, “Cancer” option is selected in the disease diagnosis question.	SOEP ^47^
No comorbidities	Baseline category. Dummy variable coded 1 if the individual reports no comorbidities in a given year, and 0 otherwise. Respectively, “No Disease” option is selected in the disease diagnosis question.	SOEP ^47^
1 comorbidity	Dummy variable coded 1 if the individual reports 1 comorbidity in a given year, and 0 otherwise. Respectively, 1 of the following is selected in the disease diagnosis question: “Diabetes”, “Asthma”, “Cardiac disease”, “Stroke”, “Migraine”, “Hypertension”, “Depression”, “Dementia”, “Other”.	SOEP ^47^
2 comorbidities	Dummy variable coded 1 if the individual reports 2 comorbidities in a given year, and 0 otherwise. Respectively, 2 of the following is selected in the disease diagnosis question: “Diabetes”, “Asthma”, “Cardiac disease”, “Stroke”, “Migraine”, “Hypertension”, “Depression”, “Dementia”, “Other”.	SOEP ^47^
3 comorbidities	Dummy variable coded 1 if the individual reports 3 comorbidities in a given year, and 0 otherwise. Respectively, 3 of the following is selected in the disease diagnosis question: “Diabetes”, “Asthma”, “Cardiac disease”, “Stroke”, “Migraine”, “Hypertension”, “Depression”, “Dementia”, “Other”.	SOEP ^47^
4 comorbidities	Dummy variable coded 1 if the individual reports 4 comorbidities in a given year, and 0 otherwise. Respectively, 4 of the following is selected in the disease diagnosis question: “Diabetes”, “Asthma”, “Cardiac disease”, “Stroke”, “Migraine”, “Hypertension”, “Depression”, “Dementia”, “Other”.	SOEP ^47^
5 comorbidities	Dummy variable coded 1 if the individual reports 5 comorbidities in a given year, and 0 otherwise. Respectively, 5 of the following is selected in the disease diagnosis question: “Diabetes”, “Asthma”, “Cardiac disease”, “Stroke”, “Migraine”, “Hypertension”, “Depression”, “Dementia”, “Other”.	SOEP ^47^
Gender	Dummy variable coded 1 if the individual is a woman, and 0 otherwise.	SOEP ^47^
Household head	Baseline category. Dummy variable coded 1 if the individual is the household head in a given year, and 0 otherwise.	SOEP ^47^
Partner	Dummy variable coded 1 if the individual is the partner of the household head in a given year, and 0 otherwise.	SOEP ^47^
Child	Dummy variable coded 1 if the individual is the child of the household head in a given year, and 0 otherwise.	SOEP ^47^
Relative	Dummy variable coded 1 if the individual is a relative of the household head in a given year, and 0 otherwise.	SOEP ^47^
Non‐relative	Dummy variable coded 1 if the individual is not a relative of the household head in a given year, and 0 otherwise.	SOEP ^47^
Age	Age of the individual in a given year.	SOEP ^47^
Less than high school	Baseline category. Dummy variable coded 1 if the highest education level reported by the individual is less than high school in a given year, and 0 otherwise.	SOEP ^47^
High school	Dummy variable coded 1 if the highest education level reported by the individual is high school in a given year, and 0 otherwise.	SOEP ^47^
More than high school	Dummy variable coded 1 if the highest education level reported by the individual is more than high school in a given year, and 0 otherwise.	SOEP ^47^
Working	Dummy variable coded 1 if the individuals reports to be working in a given year, and 0 otherwise.	SOEP ^47^

## APPENDIX 2

### Descriptive Statistics

**Table:**

Variable	Observations	Mean	Standard Deviation	Minimum	Maximum
Job income (log)	109,565	6.36	4.75	0	14.04
Work status	121,245	2.19	0.86	1	3
Working hours (log)	121,245	3.79	3.68	0	8.85
Pension (log)	25,720	9.43	0.81	3.56	12.80
Non‐cancer control	84,567	0.95	0.22	0	1
Before cancer diagnosis	84,567	0.01	0.12	0	1
Cancer diagnosis	84,567	0.04	0.18	0	1
No comorbidities	86,404	0.55	0.50	0	1
1 comorbidity	86,404	0.26	0.44	0	1
2 comorbidities	86,404	0.12	0.33	0	1
3 comorbidities	86,404	0.05	0.22	0	1
4 comorbidities	86,404	0.01	0.10	0	1
5 comorbidities	86,404	0.00	0.04	0	1
Female	161,367	0.52	0.50	0	1
Household head	161,369	0.39	0.49	0	1
Partner	161,369	0.25	0.43	0	1
Child	161,369	0.34	0.47	0	1
Relative	161,369	0.01	0.11	0	1
Non‐relative	161,369	0.01	0.08	0	1
Age	160,509	36.89	22.82	0	105
Less than high school	103,691	0.17	0.37	0	1
High school	103,691	0.61	0.49	0	1
More than high school	103,691	0.23	0.42	0	1
Working	107,662	0.60	0.49	0	1

## APPENDIX 3

### Correlation Tables for Whole Sample and Working Population Sample

**Table:**

	Cancer diagnosis	Comorbidities	Gender	Household position	Age	Education	Occupation
Cancer diagnosis status	1.00						
Comorbidities	0.11	1.00					
Gender	0.02	−0.03	1.00				
Household position	−0.04	−0.15	0.13	1.00			
Age	0.15	0.48	−0.01	−0.31	1.00		
Education	0.02	−0.06	−0.10	−0.14	0.02	1.00	
Work status	0.11	0.34	0.08	−0.01	0.47	−0.16	1.00
*N* = 81,232							

## References

[1] WHO . World Health Statistics 2016: Monitoring Health for the Sustainable Development Goals (SDGs). Geneva: WHO; 2018.

[2] Statistisches Bundesamt . Gesundheit: Todesursachen in Deutschland 2015. Wiesbaden; 2017.

[3] Azzani M , Roslani AC , Su TT . The perceived cancer‐related financial hardship among patients and their families: a systematic review. Support Care Cancer. 2015;23(3):889‐898. 25337681 10.1007/s00520-014-2474-y

[4] de Souza JA , Wong Y‐N . Financial distress in cancer patients. J Med Person. 2013;11(2):73‐77. 10.1007/s12682-013-0152-3 PMC3859450 24349677

[5] Meropol NJ , Schulman KA . Cost of cancer care: issues and implications. J Clin Oncol. 2007;25(2):180‐186. 17210937 10.1200/JCO.2006.09.6081

[6] Zafar SY , Peppercorn JM , Schrag D , et al. The financial toxicity of cancer treatment: a pilot study assessing out‐of‐pocket expenses and the insured cancer patient's experience. Oncologist. 2013;18(4):381‐390. 23442307 10.1634/theoncologist.2012-0279 PMC3639525

[7] Altice CK , Banegas MP , Tucker‐Seeley RD , Yabroff KR . Financial hardships experienced by cancer survivors: a systematic review. JNCI: J Natl Cancer Inst. 2017;109(2):1‐17. 10.1093/jnci/djw205 PMC6075571 27754926

[8] Bestvina CM , Zullig LL , Yousuf ZS . The implications of out‐of‐pocket cost of cancer treatment in the USA: a critical appraisal of the literature. Future Oncol. 2014;10(14):2189‐2199. 25471033 10.2217/fon.14.130

[9] Gordon LG , Merollini KM , Lowe A , Chan RJ . A systematic review of financial toxicity among cancer survivors: we can’t pay the co‐pay. The Patient‐Patient‐Centered Outcomes Research. 2017;10(3):295‐309. 27798816 10.1007/s40271-016-0204-x

[10] Hanratty B , Holland P , Jacoby A , Whitehead M . Financial stress and strain associated with terminal cancer—a review of the evidence. Palliat Med. 2007;21(7):595‐607. 17942498 10.1177/0269216307082476

[11] Witte J , Mehlis K , Surmann B , et al. Methods for measuring financial toxicity after cancer diagnosis and treatment: a systematic review and its implications. Ann Oncol. 2019;30(7):1061‐1070. 31046080 10.1093/annonc/mdz140 PMC6637374

[12] Mehlis K , Witte J , Surmann B , et al. The patient‐level effect of the cost of cancer care–financial burden in German cancer patients. BMC Cancer. 2020;20:1‐8. 10.1186/s12885-020-07028-4 PMC7275553 32503459

[13] Apostolidis L , Mehlis K , Witte J , et al. Financial toxicity in patients with colorectal cancer and neuroendocrine tumors. American Society of Clin Oncol (R Coll Radiol). 2018;36(15_suppl):6533.

[14] Bikowski K . Wirtschafliche Auswrikungen einer Krebserkrankung. Unveröffentlichte Untersuchung am Nationalen Centrum für Tumorerkrankungen Heidelberg 2009.

[15] Büttner M , König H‐H , Löbner M , et al. Out‐of‐pocket‐payments and the financial burden of 502 cancer patients of working age in Germany: results from a longitudinal study. Support Care Cancer. 2019;27(6):2221‐2228. 30315427 10.1007/s00520-018-4498-1

[16] Dietsche S . Editor Risk‐factors for poverty following cancer. Oncology Research and Treatment; 2018: Karger Allschwilerstrasse 10, CH‐4009 Basel, Switzerland.

[17] Mehlis K , Witte J , Surmann B , et al. Financial toxicity in cancer patients: impact of a chronic disease on patients’ economic situation and psychosocial outcomes. PPmP‐Psychotherapie· Psychosomatik· Medizinische Psychologie. 2018;68(08):e18.

[18] Winkler EC , Mehlis K , Surmann B , et al. Financial toxicity in German cancer patients: how does a chronic disease impact the economic situation? Ann Oncol. 2018;29(suppl_8):mdy424. 079.

[19] Witte J , Mehlis K , Kudlich M , et al. Subjective financial burden among German cancer patients‐relationship of the patients’ economic situation and subjective distress. Value in Health. 2017;20(9):A457.

[20] Busse R , Blümel M , Knieps F , Bärnighausen T . Statutory health insurance in Germany: a health system shaped by 135 years of solidarity, self‐governance, and competition. The Lancet. 2017;390(10097):882‐897. 10.1016/S0140-6736(17)31280-1 28684025

[21] Obermann K , Müller P , Müller H‐H , Glazinski B . The German Health Care System: Accesing the German Health Care Market. Heidelberg: mednochzwei Verlag GmbH; 2016.

[22] Cherny N , Sullivan R , Torode J , Saar M , Eniu A . ESMO European consortium study on the availability, out‐of‐pocket costs and accessibility of antineoplastic medicines in Europe. Ann Oncol. 2016;27(8):1423‐1443. 27457309 10.1093/annonc/mdw213

[23] Kandolf Sekulovic L , Peris K , Hauschild A , et al. More than 5000 patients with metastatic melanoma in Europe per year do not have access to recommended first‐line innovative treatments. Eur J Cancer. 2017;75:313‐322. 28264791 10.1016/j.ejca.2017.01.012

[24] Walther J . Krebs und Armut. Forum. Springer; 2011.

[25] Freund MK . Volker; Faber, Gerhard; Seifart. Gesundheitspolitische Schriftenreihe: Ulf. Finanzielle und soziale Folgen der Krebserkrankung für junge Menschen; 2019.

[26] Gruber U , FEGG M , Buchmann M , KOLB H‐J , Hiddemann W . The long‐term psychosocial effects of haematopoetic stem cell transplantation. Eur J Cancer Care. 2003;12(3):249‐256. 12919304

[27] Hensel M , Egerer G , Schneeweiss A , Goldschmidt H , Ho A . Quality of life and rehabilitation in social and professional life after autologous stem cell transplantation. Ann Oncol. 2002;13(2):209‐217. 11885996 10.1093/annonc/mdf031

[28] Heuser C , Halbach S , Kowalski C , Enders A , Pfaff H , Ernstmann N . Sociodemographic and disease‐related determinants of return to work among women with breast cancer: a German longitudinal cohort study. BMC Health serv Res. 2018;18(1):1000. 30594181 10.1186/s12913-018-3768-4 PMC6311058

[29] Noeres D , Park‐Simon T‐W , Grabow J , et al. Return to work after treatment for primary breast cancer over a 6‐year period: results from a prospective study comparing patients with the general population. Support Care Cancer. 2013;21(7):1901‐1909. 23417517 10.1007/s00520-013-1739-1

[30] Arndt V , Koch‐Gallenkamp L , Bertram H , et al. Return to work after cancer. A multi‐regional population‐based study from Germany. Acta Oncol. 2019;58(5):811‐818. 30777496 10.1080/0284186X.2018.1557341

[31] Mehnert A , Koch U . Predictors of employment among cancer survivors after medical rehabilitation‐a prospective study. Scand J Work Environ Health. 2013;39(1):76‐87. 22422040 10.5271/sjweh.3291

[32] Ullrich A , Rath HM , Otto U , et al. Return to work in prostate cancer survivors–findings from a prospective study on occupational reintegration following a cancer rehabilitation program. BMC Cancer. 2018;18(1):751. 30029637 10.1186/s12885-018-4614-0 PMC6053748

[33] Geue K , Sender A , Schmidt R , et al. Gender‐specific quality of life after cancer in young adulthood: a comparison with the general population. Qual Life Res. 2014;23(4):1377‐1386. 24197479 10.1007/s11136-013-0559-6

[34] Leuteritz K , Friedrich M , Sender A , Nowe E , Stoebel‐Richter Y , Geue K . Life satisfaction in young adults with cancer and the role of sociodemographic, medical, and psychosocial factors: results of a longitudinal study. Cancer. 2018;124(22):4374‐4382. 30198085 10.1002/cncr.31659

[35] Schaefer R , Schlander M . Is the National Institute for Health and Care Excellence (NICE) in England more ‘innovation‐friendly’than the Federal Joint Committee (G‐BA) in Germany? Expert Rev Pharmacoecon & Outcomes Res. 2019;19(4):453‐462. 30556745 10.1080/14737167.2019.1559732

[36] Goebel J , Grabka MM , Liebig S , et al. The German socio‐economic panel (SOEP). Jahrbücher für Nationalökonomie und Statistik. 2019;239(2):345‐360.

[37] Andersen I , Kolodziejczyk C , Thielen K , Heinesen E , Diderichsen F . The effect of breast cancer on personal income three years after diagnosis by cancer stage and education: a register‐based cohort study among Danish females. BMC Public Health. 2015;15(1):50. 25636370 10.1186/s12889-015-1387-0 PMC4320549

[38] Jensen LS , Overgaard C , Bøggild H , et al. The long‐term financial consequences of breast cancer: a Danish registry‐based cohort study. BMC Public Health. 2017;17(1):853. 29084512 10.1186/s12889-017-4839-x PMC5661907

[39] Syse A , Tønnessen M . Cancer's unequal impact on incomes in Norway. Acta Oncol. 2012;51(4):480‐489. 22150076 10.3109/0284186X.2011.640710

[40] Syse A , Tretli S , Kravdal Ø . Cancer’s impact on employment and earnings—a population‐based study from Norway. J Cancer Surviv. 2008;2(3):149‐158. 18792789 10.1007/s11764-008-0053-2

[41] Eaker S , Wigertz A , Lambert PC , et al. Breast cancer, sickness absence, income and marital status. A study on life situation 1 year prior diagnosis compared to 3 and 5 years after diagnosis. PLoS One. 2011;6(3):e18040. 21479209 10.1371/journal.pone.0018040 PMC3068139

[42] Zajacova A , Dowd JB , Schoeni RF , Wallace RB . Employment and income losses among cancer survivors: estimates from a national longitudinal survey of American families. Cancer. 2015;121(24):4425‐4432. 26501494 10.1002/cncr.29510 PMC4670608

[43] Hopkins R , Goeree R , Longo C . Estimating the national wage loss from cancer in Canada. Current Oncology. 2010;17(2):40. 20404977 10.3747/co.v17i2.477 PMC2854636

[44] Pearce A , Tomalin B , Kaambwa B , et al. Financial toxicity is more than costs of care: the relationship between employment and financial toxicity in long‐term cancer survivors. J Cancer Surviv. 2019;13(1):10‐20. 30357537 10.1007/s11764-018-0723-7

[45] Luengo‐Fernandez R , Leal J , Gray A , Sullivan R . Economic burden of cancer across the European Union: a population‐based cost analysis. Lancet Oncol. 2013;14(12):1165‐1174. 24131614 10.1016/S1470-2045(13)70442-X

[46] Roelen CA , Koopmans PC , Groothoff JW , van der Klink JJ , Bültmann U . Sickness absence and full return to work after cancer: 2‐year follow‐up of register data for different cancer sites. Psycho‐oncology. 2011;20(9):1001‐1006. 20672244 10.1002/pon.1820

[47] Socio‐Economic Panel . Data for years 2009‐2015 [Internet]. 2018.

